# Selective Membrane Redistribution and Depletion of Gα_q_-Protein by *Pasteurella multocida* Toxin

**DOI:** 10.3390/toxins8080233

**Published:** 2016-08-01

**Authors:** Nathan C. Clemons, Shuhong Luo, Mengfei Ho, Brenda A. Wilson

**Affiliations:** Department of Microbiology, University of Illinois at Urbana-Champaign, Urbana, IL 61801, USA; nclemons@illinois.edu (N.C.C.); sluo815@gmail.com (S.L.); mengho@life.illinois.edu (M.H.)

**Keywords:** dermonecrosis, *Pasteurella multocida*, G-protein downregulation, mitogen, deamidation

## Abstract

*Pasteurella multocida* toxin (PMT), the major virulence factor responsible for zoonotic atrophic rhinitis, is a protein deamidase that activates the alpha subunit of heterotrimeric G proteins. Initial activation of G alpha-q-coupled phospholipase C-beta-1 signaling by PMT is followed by uncoupling of G alpha-q-dependent signaling, causing downregulation of downstream calcium and mitogenic signaling pathways. Here, we show that PMT decreases endogenous and exogenously expressed G alpha-q protein content in host cell plasma membranes and in detergent resistant membrane (DRM) fractions. This membrane depletion of G alpha-q protein was dependent upon the catalytic activity of PMT. Results indicate that PMT-modified G alpha-q redistributes within the host cell membrane from the DRM fraction into the soluble membrane and cytosolic fractions. In contrast, PMT had no affect on G alpha-s or G beta protein levels, which are not substrate targets of PMT. PMT also had no affect on G alpha-11 levels, even though G alpha-11 can serve as a substrate for deamidation by PMT, suggesting that membrane depletion of PMT-modified G-alpha-q has specificity.

## 1. Introduction

*Pasteurella multocida* toxin (PMT) is the major virulence factor responsible for atrophic rhinitis, pasteurellosis, and dermonecrosis caused by infection with toxigenic capsular type D and some A strains of *Pasteurella multocida* (reviewed in [1]). PMT is a 1285-amino acid AB protein toxin that acts intracellularly by gaining entry into host cells via binding membrane phospholipids phosphatidylcholine, sphingomyelin, and an as-yet unknown trypsin-sensitive protein partner [2]. PMT is subsequently endocytosed and released from the late endosomes into the cytosol in a pH dependent manner [3,4,5], where it targets heterotrimeric G-proteins and modulates their downstream signaling pathways (reviewed in [6]). PMT acts on α subunits of the Gα_q/11_, Gα_12/13_, and Gα*_i_*_1/2/3_, but not Gα_s_ families [7,8,9,10] through deamidation of a specific Gln residue in the switch II region of the active site of Gα subunits [11], changing Glu to Gln and converting the Gα-protein into a constitutively active form.

Physiologically, PMT stimulates phospholipase C β-1 (PLCβ1) signaling through its preferential activation of Gα_q_ of the Gα_q/11_ family of G-proteins [9,10]. By doing so, PMT increases the intracellular cytoplasmic concentration of Ca^2+^ and activates mitogenic signaling pathways, leading to increased cellular proliferation and in some cell types cell differentiation [9,12]. PMT also indirectly activates Rho signal transduction through its action on Gα_q/11_ and Gα_12/13_ [13,14,15,16], leading to actin cytoskeletal rearrangements and changes in cellular morphology [10,14,17,18,19,20,21]. Thus far, the only Gα subunit that PMT does not deamidate or activate is Gα_s_ [8,22,23], the stimulatory regulator of adenylate cyclase (AC). Instead, PMT inhibits adenylate cyclase activity through its activation of inhibitory AC regulators of the Gα*_i_* family [23]. PMT activation of these different G-protein signaling pathways, in conjunction with crosstalk among the downstream signaling pathways, can lead to multiple cellular outcomes (reviewed in [6,24]).

Although PMT modulates numerous intracellular signaling pathways to dictate cellular fate by constitutive activation of the Gα proteins, the overall activation of downstream signaling is not sustained and is subsequently mitigated by an unknown mechanism of downregulation. PMT causes an initial strong activation of downstream calcium and mitogenic signaling, which is subsequently followed by an uncoupling of the signaling in the case of Gα_q-_CPLCβ1 [9,21] and Gα*_i_*_-_AC [23]. Moreover, it has been shown that prolonged PMT treatment is sufficient to downregulate Gα_q_-mediated G-protein-coupled inward rectifying K^+^ channels [25,26,27] and to block agonist-induced binding of GTPγS binding to Gα*_i_* protein [23].

It is well established that sustained exposure of many G-protein-coupled receptors (GPCR), including those coupled with G_q_ and G_11_ proteins, to their cognate agonist results in desensitization of the pathway due to a sequestration, modification or reduction in cellular levels of the receptor and/or the Gα subunit [28,29,30,31,32,33,34]. This desensitization or downregulation may also result from a redistribution of the Gα proteins in the membrane to the cytosol, or within membranes from detergent resistant membranes (DRMs, or lipid rafts) to more soluble membrane fractions [34,35,36,37,38]. Indeed, some GPCRs, G proteins, and their effectors are not only localized in DRMs, but also dynamically move in and out of them during signal transduction [39]. In each of these cases, the downregulation of Gα signaling was mediated through agonist binding of the GPCR. Until now, discerning how the subsequent cellular downregulation of PMT-modified Gα-proteins occurs has not been studied.

In this study, we investigated the fate of the Gα_q_, Gα_11_, and Gα_s_ proteins in HEK-293T cells after PMT treatment by determining the levels of the Gα protein in whole cell lysates, total membranes, detergent resistant membranes (DRMs), and the soluble membrane and cytosolic fractions. Results from our experiments indicate that treatment of HEK-293T cells with wildtype PMT, but not catalytically inactive mutant PMT (C1165S), stimulates the selective downregulation of Gα_q_, but not Gα_s_ or Gα_11_, through redistribution of the Gα_q_ protein from DRMs to soluble membrane and cytosolic fractions with overall loss of Gα_q_ protein from the plasma membrane.

## 2. Results

### 2.1. The Cytotoxic Effect of PMT on Cultured HEK-293T Cells

**Figure 1:**
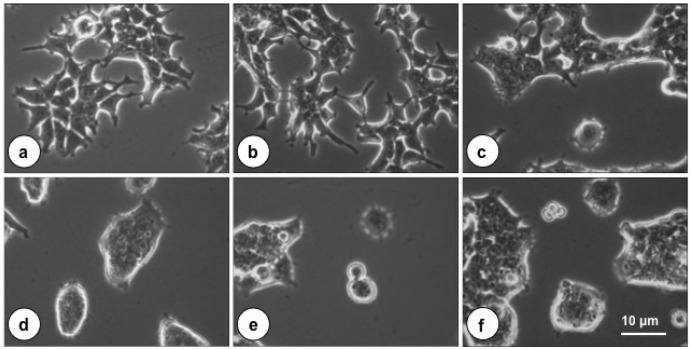
Effect of *Pasteurella* *multocida* toxin (PMT) on the morphology of HEK-293T cells. Shown are representative phase-contrast micrographs of HEK-293T cells treated overnight with varying concentrations of PMT: (**a**) no toxin; (**b**) 0.01 nM; (**c**) 0.07 nM; (**d**) 0.65 nM; (**e**) 1.6 nM; and (**f**) 3.2 nM. Bar, 10 µm.

Recombinant, full-length PMT induced morphological effects in cultured HEK-293T cells, similar to that previously described for other cells [21]. PMT caused dosed-dependent rounding up and clumping of cells, as shown in Figure 1, and reorganization of the actin cytoskeleton (data not shown). Similar treatment with *N*-terminal (residues 1–506) or *C*-terminal (residues 486–1285) PMT fragments had no effect on cell morphology (data not shown).

### 2.2. PMT Treatment Reduces Endogenous Gα_q/11_ Levels in Detergent Resistant Membranes (DRMs)

**Figure 2:**
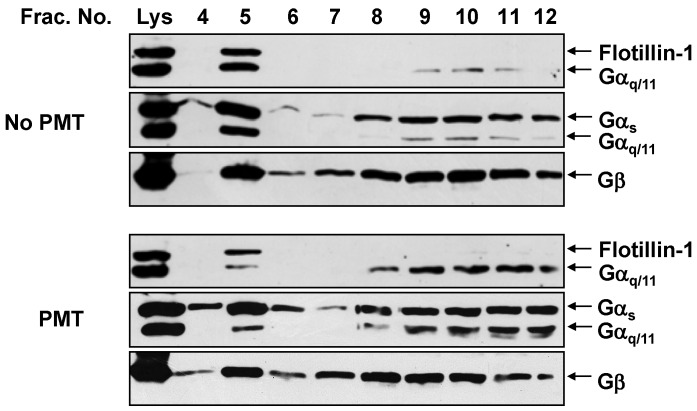
PMT-mediated downregulation of endogenous Gα_q/11_ levels in detergent resistant membranes (DRMs). HEK-293T cells were incubated without or with 0.3 nM PMT for 10 h. Preparation of cell lysates and fractionation by OptiPrep density gradient centrifugation was performed, as described in Methods Section. Each sample was separated into 121-mL fractions from low density (top, fraction #1) to high density (bottom, fraction #12), with fraction #5 containing the DRMs, as evidenced by the presence of the DRM-specific marker flotillin-1. The proteins were resolved by 10% SDS-PAGE gel and subsequently analyzed by immunoblotting using antibodies against Gα_q/11_, Gα_s_, Gβ, and flotillin-1 present in the cell lysates (Lys) and fractions #4–12 from the density gradient.

Because PMT acts directly on its Gα substrates and subsequent modulation of signaling is independent of agonist-receptor coupling to the G protein [9,40,41], we considered two possible mechanisms for PMT-mediated down-regulation of Gα_q/11_-PLCβ1 signaling: (1) PMT deamidation and activation of Gα_q_ and/or Gα_11_ leads to degradation of the resulting constitutively active Gα subunit and loss of cellular levels of Gα_q_ and/or Gα_11_; or (2) PMT deamidation and activation of Gα_q_ and/or Gα_11_ leads to redistribution of the resulting constitutively active Gα subunit from DRMs to soluble membrane and/or cytosolic fractions. In agreement with a previous report [42], immunoblot analysis of total cell lysates using antibodies against the *C*-terminus of Gα_q/11_ showed that overnight treatment of the cells with PMT did not alter endogenous protein levels of Gα_q/11_ in total cell lysates (Figure 2). This result did not support the first proposed mechanism for PMT-mediated downregulation of Gα_q/11_ signaling, and so we next considered the second possibility that the downregulation of signaling might result from loss of the PMT-activated Gα_q/11_ from the membrane. Thus, we examined whether PMT treatment could cause loss of Gα_q/11_ protein from detergent-resistant membranes (DRMs, also called lipid rafts) since many membrane signaling molecules are enriched in these membrane micro-domains.

PMT-treated HEK-293T cells were lysed in the presence of detergent and the cellular contents were subjected to subcellular fractionation by separation using Optiprep density gradient centrifugation, followed by immunoblotting analysis of the gradient fractions. In the density gradient centrifugation profile, fractions #1–7 contain low-density lipid-rich membranes and vesicles, while fractions #8–12 contain high-density protein-rich soluble membrane and cytosolic fractions [43]. As shown in Figure 2, whereas the abundant Gβ and Gα_s_ subunits were found throughout the gradient in both higher density (#8–12) and lower density fractions (#4–7) from untreated control cells, the majority of the less abundant Gα_q/11_ subunits were localized mostly to the low-density, DRM-containing fraction (#5), as evidenced by its co-localization with flotillin-1, a known specific marker for DRMs [44]. In contrast, PMT treatment resulted in the depletion of Gα_q/11_, but not Gβ or Gα_s_, from the DRM-containing fraction #5. With this loss was a concomitant increase of Gα_q/11_ in the soluble membrane and cytosolic fractions (#8–12), suggesting that PMT modification and activation of Gα_q/11_ results in the release of active form of the Gα_q/11_ protein from DRMs. That PMT treatment did not significantly alter the levels of Gα_s_ or Gβ in DRMs, as compared with flotillin-1, confirms previous findings, indicating that Gα_s_ protein is not a target substrate of PMT.

**Figure 3:**
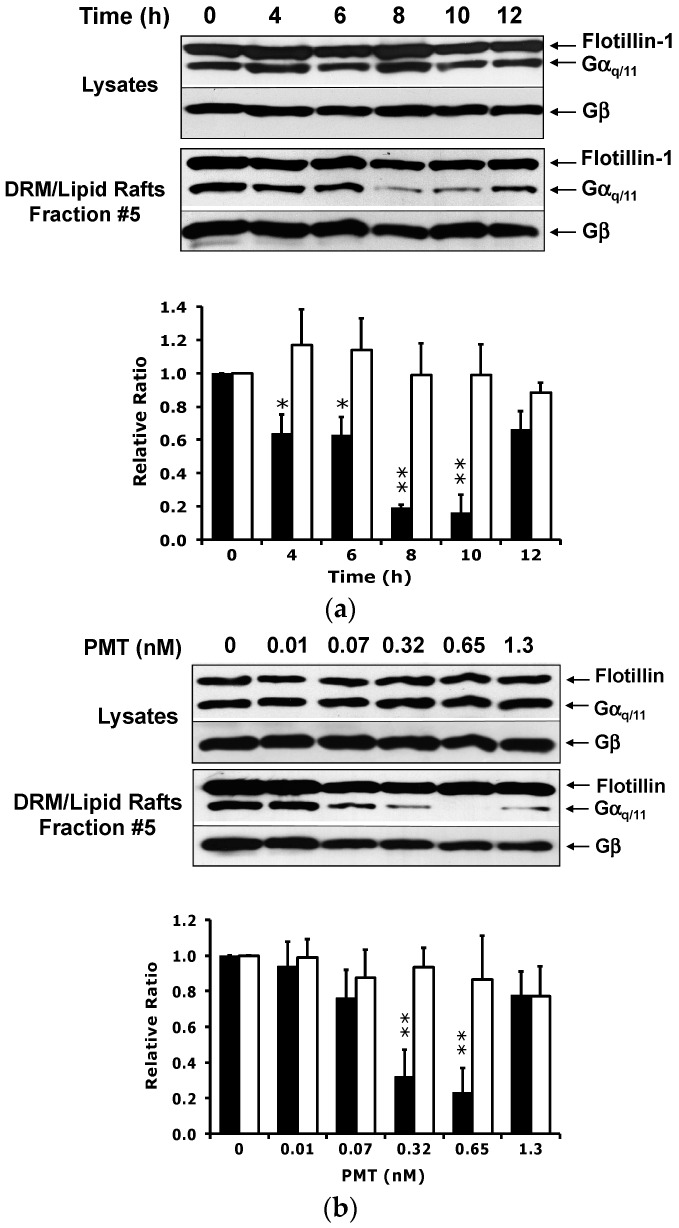
Time course and dose–response of PMT-mediated downregulation of endogenous Gα_q/11_ levels in detergent resistant membranes (DRMs). (**a**) Time course of PMT-mediated downregulation of Gα_q/11_ levels in DRMs. HEK-293T cells were incubated with 0.3 nM PMT for the indicated times. (**b**) Dose–response of PMT-mediated downregulation of Gα_q/11_ levels in DRMs. HEK-293T cells were incubated with the indicated concentrations of PMT for 10 h. Shown are representative immunoblots of the lysates and the DRM-containing fraction #5 of the density gradients, prepared, separated by density gradient fractionation, and analyzed by immunoblotting, as described in Figure 2. Shown below the immunoblots are the corresponding plots of the quantification of the protein bands, where the data are presented as the mean ± SD of the relative ratio of the intensity of the Gα_q/11_ or Gβ bands to the corresponding flotillin-1 bands. Filled bars: Relative ratio of Gα_q/11_/flotillin-1. Open bars: Relative ratio of Gβ/flotillin-1. * *p* < 0.01; ** *p* < 0.001, PMT treatment vs. no treatment for each blot (unpaired two-tailed Student’s *t*-test). All data are from three or more independent experiments.

A time course revealed that while overall endogenous Gα_q/11_ protein levels in cells were not discernably affected by exposure to PMT over 12 h, the Gα_q/11_ levels in DRMs were reduced by over 80% by 8–10 h in cells treated with 0.3 nM PMT (Figure 3a). This result supports the notion that PMT action on Gα_q/11_ causes a redistribution of the protein, and not degradation within this timeframe. By 12 h, however, the Gα_q/11_ levels in DRMs were restored to nearly 80% that of untreated cells. Again, Gβ and flotillin levels remained unaffected by PMT treatment. The decrease in Gα_q/11_ levels in DRMs was also dose-dependent, exhibiting an EC_50_ value of ~70 pM (Figure 3b). Although the loss of Gα_q/11_ from DRMs was about 65% and 75% after 10 h of PMT treatment at a concentration of 320 pM and 650 pM, respectively, the Gα_q/11_ levels were again restored at higher PMT concentrations of 1.3 nM. Similar results were obtained using Swiss 3T3 cells (data not shown). Taken together, PMT selectively induces the loss of Gα_q/11_ proteins from DRMs in a time and dose dependent manner, although this loss can be masked or overcome at later times or higher toxin concentrations by PMT-stimulation of new protein synthesis.

### 2.3. PMT Treatment Depletes Gα_q_, but Not Gα_11_, Protein Levels in DRMs and the Plasma Membrane

**Figure 4:**
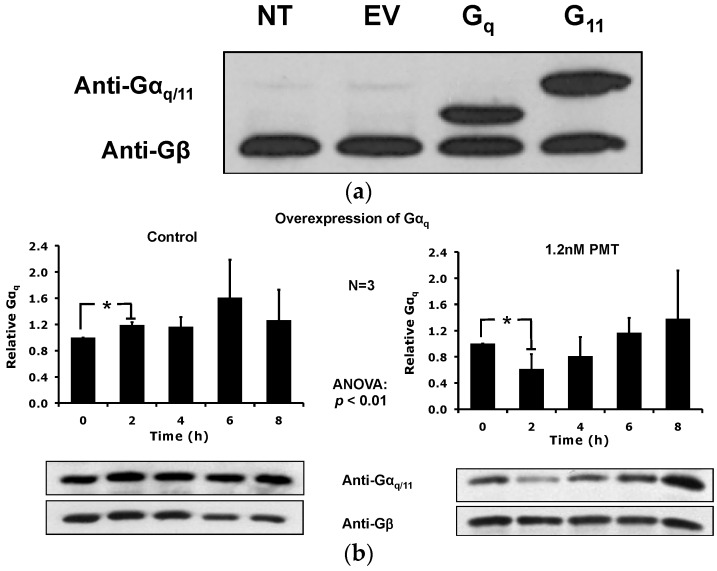

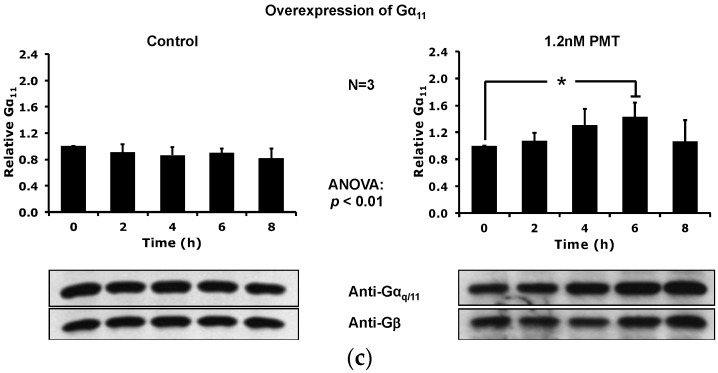
PMT-mediated downregulation of Gα_q_ and Gα_11_ levels in detergent resistant membranes (DRMs) from HEK-293T cells overexpressing Gα_q_ or Gα_11_. (**a**) Shown is a representative immunoblot of whole cell membranes from HEK-293T cells transfected for 24 h with either no transfection (NT), pcDNA3.1/Gα_q_, pcDNA3.1/Gα_11_, or empty vector pcDNA3.1 (EV). The proteins were resolved by 10% SDS-PAGE gel and subsequently analyzed by immunoblotting using antibodies against Gα_q/11_ and Gβ. (**b**) Time course of PMT-treated HEK-293T cells overexpressing Gα_q_. (**c**) Time course of PMT-treated HEK-293T cells overexpressing Gα_11_. Shown are representative immunoblots (bottom panels) and the corresponding quantification plots (top panels) of DRMs, prepared using sucrose density gradient fractionation as described in Methods, and analyzed as described in Figure 2. Cells overexpressing Gα_q_ or Gα_11_ were treated without PMT (left panel) or with 1.2 nM PMT (right panel) for the indicated times. Proteins in the DRM-containing fraction #5 of the density gradient were resolved by 10% SDS-PAGE gel and analyzed by immunoblotting using antibodies against Gα_q/11_ or Gβ. Plotted data are presented as the mean ± SD. Filled bars: relative ratio of Gα_q/11_/Gβ. *N* is the number of independent experiments performed. * *p* < 0.05, PMT treatment vs. time 0 h for each blot.

To distinguish the specificity of the PMT-mediated DRM depletion for Gα_q_ and Gα_11_, we expressed exogenously either Gα_q_ or Gα_11_ in HEK-293T cells over the endogenous background and detected the proteins using the anti-Gα_q/11_ antibodies under conditions where the corresponding endogenous proteins were negligible (Figure 4a). The transfected cells overexpressing Gα_q_ or Gα_11_ were then used to determine the effect of PMT treatment on the DRM levels of overexpressed Gα_q_ or Gα_11_ proteins vs. Gβ. Results of immunoblot analysis of the DRM-containing fractions from a time course of treatment with 1.2 nM PMT revealed that PMT treatment caused a transient DRM depletion of Gα_q_ (Figure 4b) but not Gα_11_ (Figure 4c) after 2 h treatment.

**Figure 5:**
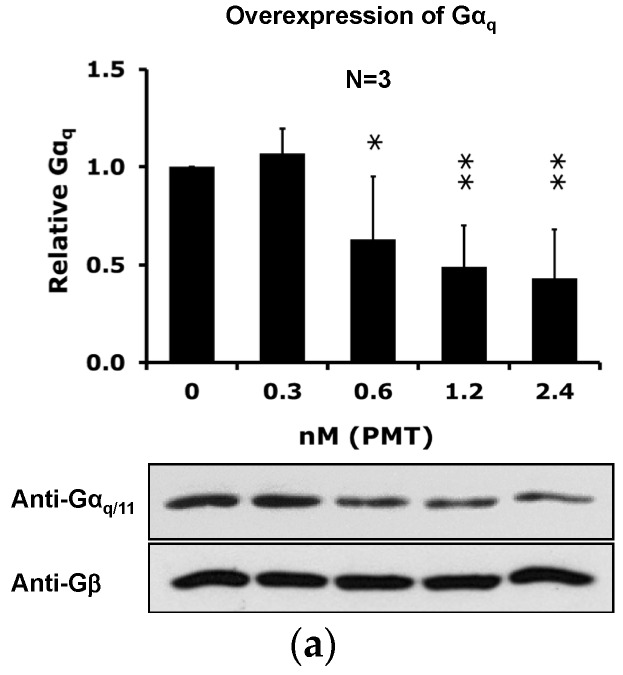

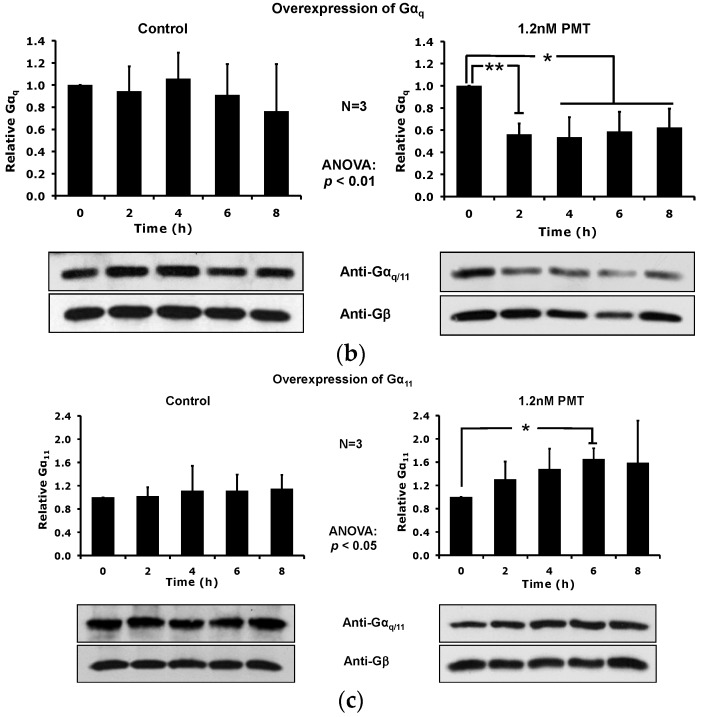
PMT-mediated downregulation of Gα_q_ and Gα_11_ levels in membrane preparations from HEK-293T cells overexpressing Gα_q_ or Gα_11_. (**a**) Dose course of PMT-mediated downregulation of Gα_q_ levels in membrane preparations from HEK-293T cells overexpressing Gα_q_ treated with the indicated dosages of PMT for 3 h. (**b**) Time course of PMT-mediated downregulation of Gα_q_ levels in membrane preparations from HEK-293T cells overexpressing Gα_q_ treated without PMT (left panels) or with 1.2 nM PMT (right panels) for the indicated times. (**c**) Time course of PMT-mediated downregulation of Gα_11_ levels in whole membrane preparations from HEK-293T cells overexpressing Gα_11_ treated without or with PMT, as in (**b**). Shown are representative immunoblots (bottom panels) and the corresponding quantification plots (top panels) of whole cell membranes, prepared and analyzed as described in the Methods Section. The proteins in the membrane fractions were resolved by 10% SDS-PAGE gel and subsequently analyzed by immunoblotting using antibodies against Gα_q/11_ and Gβ. Filled bars: Relative ratio of Gα_q/11_/Gβ. *N* is the number of independent experiments performed. * *p* < 0.05, ** *p* < 0.01, PMT treatment vs. time 0 h or dose 0 for each blot.

Considering that PMT selectively depletes Gα_q_ and not Gα_11_, we considered the possibility that one reason we did not observe discernable depletion of endogenous Gα_q_ protein levels from total cell lysates (Figure 2 and Figure 3) might be due to masking by the remaining Gα_11_ present in the membranes, as well as by the replacement with newly synthesized protein. We thus investigated the effect of PMT on Gα_q_ and Gα_11_ levels in total membrane preparations from cells selectively overexpressing each of the proteins. As shown in Figure 5, PMT treatment caused a dose- and time-dependent reduction of Gα_q_, but not Gα_11_, protein levels in total membrane preparations from cells overexpressing the respective Gα protein. In contrast, PMT treatment of cells overexpressing Gα_11_ resulted in a slight increase in overall Gα_11_ protein levels in membranes. However, unlike the case for DRMs, the loss of Gα_q_ from membranes did not recover over the 8 h treatment period.

**Figure 6:**
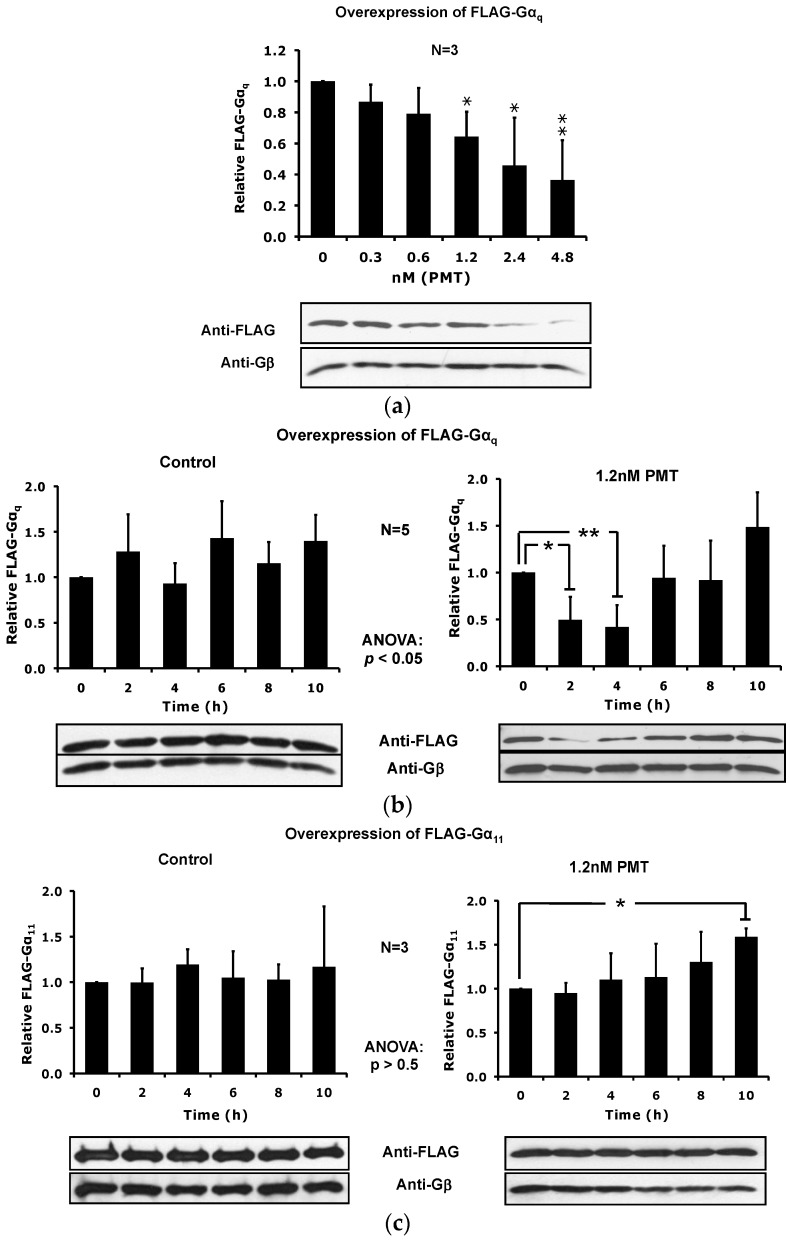
PMT-mediated downregulation of FLAG-Gα_q_ and FLAG-Gα_11_ protein levels in detergent resistant membranes (DRMs) from HEK-293T cells overexpressing FLAG-Gα_q_ or FLAG-Gα_11_. (**a**) Dose-response of PMT-treated HEK-293T cells overexpressing FLAG-Gα_q_ were treated with the indicated dosages of PMT for 3 h. (**b**) Time course of PMT-treated HEK-293T cells overexpressing FLAG-Gα_q_ were treated without (left panel) or with 1.2 nM PMT (right panel) for the indicated times. (**c**) Time course of PMT-treated HEK-293T cells overexpressing FLAG-Gα_11_ treated with PMT, as in (**b**). Shown are representative immunoblots (bottom panels) and the corresponding quantification plots (top panels) of DRMs, prepared using OptiPrep density gradient fractionation and analyzed as described in Figure 2. Proteins in the DRM-containing fraction #5 of the density gradient were resolved by 10% SDS-PAGE gel and analyzed by immunoblotting using antibodies against the FLAG-tag or Gβ. Plotted data are presented as the mean ± SD. Filled bars: Relative ratio of FLAG/Gβ. *N* is the number of independent experiments performed. * *p* < 0.05, ** *p* < 0.01, PMT treatment vs. time 0 h or dose 0 for each blot.

To further discern specificity for PMT-modified Gα_q_ and not Gα_11_ as preferred target for membrane depletion, we transiently overexpressed FLAG-tagged Gα_q_ or FLAG-tagged Gα_11_ separately in HEK-293T cells and investigated the effect of PMT on the corresponding protein levels in DRMs. Results shown in Figure 6 are similar to those obtained in Figure 4. PMT caused a dose- and time-dependent depletion of FLAG-Gα_q_ but not FLAG-Gα_11_ from DRMs. Again the effect was transient with FLAG-Gα_q_ levels recovering by 6–8 h (Figure 6c). Also consistent with data in Figure 4, cells overexpressing FLAG-Gα_11_ showed no PMT-mediated loss of the protein in DRMs, and instead the protein levels increased slightly (Figure 6c).

### 2.4. The Catalytic Activity of PMT Is Required for Membrane Depletion of Gα_q_

**Figure 7:**
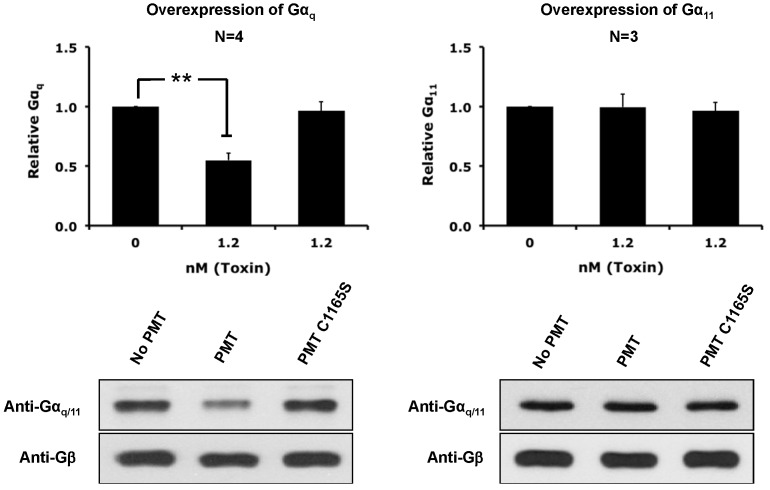
Dependence of PMT-mediated downregulation of membrane levels of Gα_q_ protein on the catalytic activity of PMT. HEK-293T cells overexpressing Gα_q_ (left panels) or Gα_11_ (right panels) were treated for 2 h without or with 1.2 nM PMT or the catalytically inactive mutant PMT C1165S. Shown are representative immunoblots (bottom panels) and the corresponding quantification plots (top panels) of whole cell membranes, prepared and analyzed as described in Figure 3. The proteins in the membrane fractions were resolved by 10% SDS-PAGE gel and subsequently analyzed by immunoblotting using antibodies against Gα_q/11_ or Gβ. Plotted data are presented as the mean ± SD. Filled bars: relative ratio of Gα_q/11_/Gβ. *N* is the number of independent experiments performed. ** *p* < 0.01, PMT treatment vs. no treatment for each blot.

Since PMT specifically induces the depletion of only Gα_q_ from the membrane even though Gα_11_ is also apparently a substrate for deamidation, we wanted to test if the deamidase activity of PMT is required for the depletion of Gα_q_ from the membrane. The catalytic domain of PMT possesses a cysteine-histidine-aspartic acid catalytic triad in the active site, where mutation of the cysteine residue into serine (PMT C1165S) reduces the enzymatic deamidase activity by 1000-fold [45]. As shown in Figure 7, treatment of cells with PMT C1165S does not result in membrane depletion of Gα_q_, and neither PMT nor PMT C1165S caused the depletion of Gα_11_, confirming that the enzymatic activity of PMT is necessary for the depletion of Gα_q_ from the host cell membrane.

### 2.5. Overexpression of the Constitutively Active Forms of Gα_q_ Alone nor in the Presence of PMT Is Sufficient to Induce Its Depletion from the Plasma Membrane

**Figure 8:**
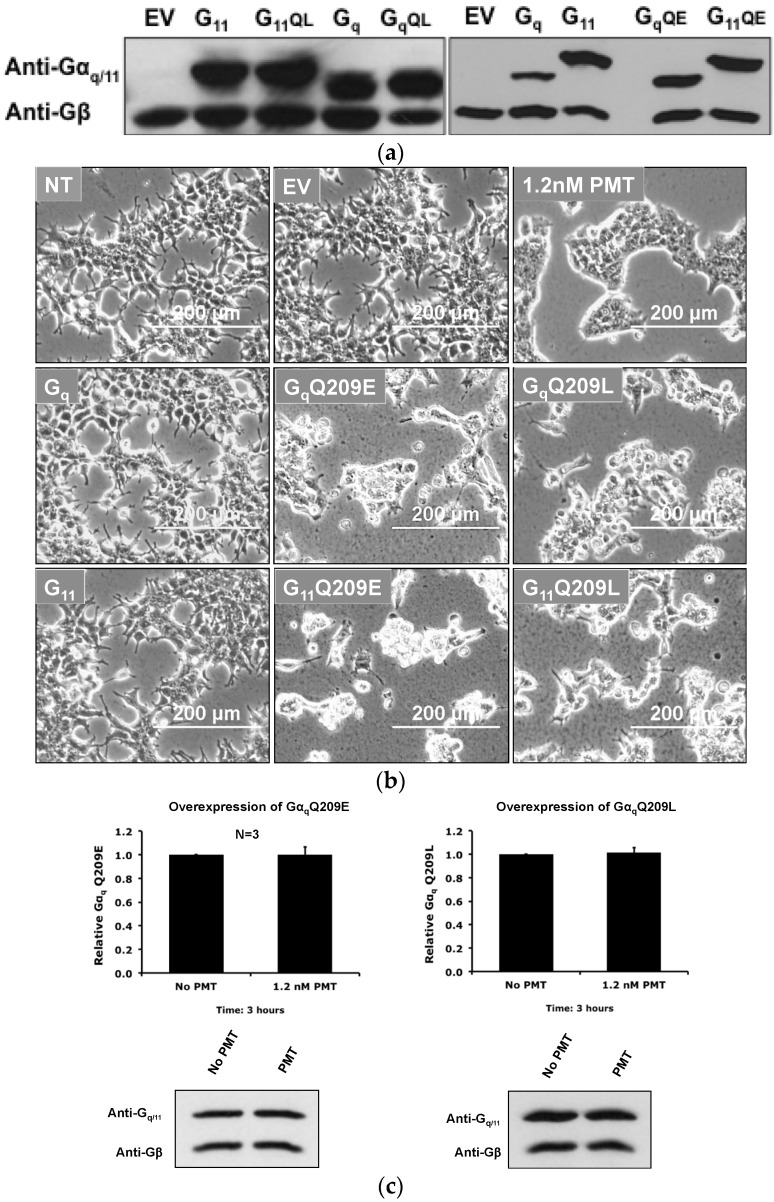
Morphology of HEK-293T cells exogenously expressing constitutively active Gα-proteins and the effect of PMT on membrane levels of constitutively active Gα_q_ proteins. (**a**) Representative immunoblots of membrane preparations of HEK-293T cells overexpressing Gα_q_, Gα_11_, or their constitutively active Q209E and Q209L counterparts, using antibodies against Gα_q/11_ or Gβ. Control membranes were from cells transfected with empty vector pcDNA3.1 (EV). (**b**) Shown are phase-contrast micrographs of HEK-293T cells overexpressing the indicated Gα-protein, or without or with 1.2 nM PMT treatment, as indicated. NT, no transfection or treatment. Bar, 200 µm. (**c**) Representative immunoblots of membrane preparations showing protein levels of Gα_q_ Q209E, Gα_q_ Q209L, or Gβ in cells without or with 1.2 nM PMT treatment for 3 h PMT. Shown above the immunoblots are the corresponding plots of the quantification of the bands, where data are presented as the mean ± SD of the relative ratios of Gα_q/11_/Gβ. *N* is number of independent experiments performed. *p* > 0.6, PMT treatment vs. no treatment for each plot.

Since the catalytic activity of PMT is required for the depletion of Gα_q_ from total membrane samples, and the consequence of PMT action renders the G-proteins constitutively active, we wanted to explore whether or not constitutive activity of Gα_q_ alone was sufficient to induce the depletion or exclusion of the activated Gα_q_ protein from the plasma membrane. We transfected HEK-293T cells separately with Gα_q_, Gα_11_, and their corresponding constitutively active mutants, with known dominant-active mutations, Gα_q_ Q209L and Gα_11_ Q209L, and with mutations equivalent to that introduced by PMT action, Gα_q_ Q209E and Gα_11_ Q209E (Figure 8a). First, we tested if overexpressing constitutively active Gα-proteins in HEK-293T cells causes similar cytotoxic morphological effects as PMT treatment. Exogenous expression of Gα_q_ or Gα_11_ caused little effect on morphology after 24 h, but, as expected, overexpression of each of the four constitutively active mutants of Gα_q_ or Gα_11_ results in the rounding up and clumping of cells, similar to that caused by PMT treatment (Figure 8b).

Unlike with the case of exogenous expression of Gα_q_ protein, PMT treatment had no affect on the localization of the exogenously expressed constitutively active Gα_q_ Q209E or Gα_q_ Q209L mutant proteins in membranes (Figure 8c), suggesting that the presence of the activated form of the Gα_q_ protein alone is not sufficient to cause its removal from the plasma membrane by PMT.

## 3. Discussion

We previously showed that microinjection of PMT into voltage-clamped *Xenopus* oocytes invoked a rapid (within 10 s) Ca^2+^-dependent Cl^−^ current, which involved activation of Gα_q/11_-PLCβ1 [9]. In this previous study, the response to PMT was transient, such that the initial strong response was followed by subsequent downregulation and unresponsiveness to further stimulation by PMT. A similar initial activation and subsequent downregulation of PMT-stimulated Ca^2+^ and mitogenic signaling was evidenced in mammalian cells through studies involving cell cycle analysis [21,46]. This downregulation of G_q_ signaling by PMT was reminiscent of receptor-mediated downregulation of G_q_-protein signaling that occurs upon prolonged exposure to agonist [34,35,36,37,38], specifically the shift of Gα_q_ proteins from DRMs to soluble membrane fractions or the cytosol during agonist-stimulated signal transduction [39]. This prompted us to explore the possibility that PMT-mediated activation of its Gα substrates, Gα_q_ and/or Gα_11_, results in a similar uncoupling of the corresponding G-protein-signaling pathway.

We considered the possibility that although PMT might have little or no effect on total cellular content of Gα_q/11_ proteins, PMT deamidation and subsequent activation of the Gα_q/11_ proteins may cause downregulation of G_q/11_ signaling through depletion of Gα_q/11_ levels in DRMs. The results presented here show for the first time that PMT induces both dose- and time-dependent depletion of Gα_q_ but not Gα_11_ from membrane lipid rafts in HEK-293T cells with concomitant shift of Gα_q_ to soluble membrane fractions or cytosol. Indeed, there was no significant decrease in Gα_q_ or Gα_11_ observed for any of the time points in the control experiments with no PMT treatment or for the Gα_11_ protein in the PMT-treated experiments; a transient decrease in Gα_q_ protein levels was observed only in PMT-treated cells. We further showed that this PMT-induced depletion occurs within DRM fractions from cells expressing endogenous levels of Gα_q/11_ proteins, as well as whole membranes and DRM fractions from cells overexpressing wildtype Gα_q_, and in DRM fractions from cells overexpressing FLAG-tagged Gα_q_, but not Gα_11_ proteins. Moreover, this PMT-mediated membrane redistribution of Gα_q_ was dependent on the deamidase activity of PMT since a catalytic mutant PMT C1165S did not cause membrane depletion of Gα_q_.

Specifically, when cellular contents of PMT treated cells were separated into fractions by OptiPrep or sucrose density gradient centrifugation, we found that PMT treatment resulted in the loss of Gα_q_ subunit from the flotillin-1-enriched DRM-containing fraction #5 with a concomitant increase of Gα_q_ protein in the more soluble fractions (#8–12) of the gradient. The effect of PMT was specific for Gα_q_, and not for other Gα subunits that are not targets of PMT, such as Gα_s_ or Gβ. Moreover, PMT-treatment caused membrane depletion of Gα_q_ and not Gα_11_. Gα_q_ and Gα_11_ share very similar structural homology and sequence identity of over 90%, and Gα_11_ can also be deamidated by PMT in cells [7,8]. The sensitive commercially available antibody that we used in this study to detect endogenous protein levels recognizes the *C*-terminus of both Gα_q_ and Gα_11_. Thus, to distinguish the specificity of the PMT-mediated DRM and membrane depletion for Gα_q_ vs. Gα_11_, we decided to use an overexpression approach, where we exogenously expressed either Gα_q_ or Gα_11_ and detected the proteins using the anti-Gα_q/11_ antibodies. Under these conditions, endogenous levels of Gα_q_ and Gα_11_ were not detectable in the resulting immunoblots.

The finding that the continued exposure to PMT was neither able to sustain the loss of nor perpetuate further loss of Gα_q_ from DRMs suggests that the cellular Gα_q_ levels in DRMs are replenished as a result of increased Gα_q_ protein synthesis. Previous studies have shown that PMT induces protein synthesis and cellular proliferation in a cycloheximide-sensitive manner [47,48]. This notion is supported by the slight increase (not decrease) observed for Gα_11_ under similar conditions. Alternatively, it is possible that the observed replenishment of Gα_q_ in the DRMs may also be due to a redistribution of the preexisting or newly synthesized monomeric Gα_q_ protein that is in the membrane. However, unlike in the case for DRMs, the loss of Gα_q_ from total membranes did not appear to replenish with time, suggesting that PMT modification and activation of Gα_q_ uncouples the signaling pathway in such a way as to interfere with the ability of newly synthesized Gα_q_ to interact with membranes, presumably through preventing reassociation with Gβγ subunits and coupling to GCPRs. The relatively faster response times for depletion and replenishment of the exogenously expressed Gα_q_ protein, compared to that observed for endogenous protein, was presumably due to the increased levels of Gα_q_ protein available as a substrate for PMT and saturation of the signaling system from enhanced protein synthesis of Gα_q_ encoded by the expression plasmid. Further, this PMT-mediated loss of Gα_q_ from DRMs and membranes is apparently masked at the total cell level (whole cell lysates) through redistribution to soluble membrane fractions or cytosol, as evidenced from the results shown in Figure 2 and Figure 3, and through synthesis of new Gα_q_ protein, which counters the overall PMT-mediated loss of Gα_q_ protein from membranes.

Since overexpression of Gα_q_ Q209E or Gα_q_ Q209L in HEK-293T cells resulted in similar morphological effects as PMT treatment, we explored whether exogenous expression of these constitutively active mutants would lead to their localization in the membrane and DRMs or would also result in their membrane depletion. Since we found that the presence of constitutively active Gα_q_ mutant proteins alone was not sufficient to induce their depletion or exclusion from membranes, we next considered whether PMT treatment could induce the removal of pre-existing constitutively active Gα_q_ from the cell plasma membrane. However, we found that PMT treatment had no effect on the membrane localization of exogenously expressed constitutively active Gα_q_ mutant proteins.

Although the antibodies we used could not distinguish whether only the deamidated form of the Gα_q_ is depleted from DRMs, our results clearly indicate the PMT catalytic activity is required. If PMT-mediated deamidation is required for depletion, and both Gα_q_ and Gα_11_ can serve as substrates for deamidation by PMT [8], then PMT-deamidated Gα_11_ should also be depleted from DRMs. However, our results (Figure 4c) indicate that this is not the case. This suggests that either cellular Gα_11_ is a very poor substrate for PMT and does not get deamidated to any significant extent during the time studied or that the as-yet-undefined factor(s) responsible for depletion do not recognize the PMT-deamidated Gα_11_ as they do the PMT-deamidated Gα_q_. These possibilities might be explored further under endogenous conditions by using antibodies that distinguish Gα_q_ from Gα_11_ coupled with those that recognize the deamidated forms of the Gα proteins, such as those previously reported [7,8].

These results suggest that PMT does not directly deplete the modified Gα_q_ protein from the membrane, but PMT might indirectly activate an unknown cellular component at the membrane or expedite a cellular process responsible for the downregulation of membrane-associated PMT-modified Gα_q_. This further suggests that the depletion mechanism of PMT-modified Gα_q_ proteins from the membrane has specificity and that PMT-catalyzed deamidation and activation of the Gα_q_ protein is the not the sole signal for initiating the downregulation process. Whether similar membrane depletion occurs for other Gα substrates of PMT once they are deamidated and activated remains to be determined.

## 4. Materials and Methods

### 4.1. Materials

Human embryonic kidney 293-T (HEK-293T) cells were obtained from the American Type Culture Collection (ATCC# CRL-3216). Goat polyclonal antibodies against Gα_s_ (A-16, sc-26766), mouse monoclonal antibody against β tubulin (D-10, sc-5274), rabbit polyclonal antibodies against Gα_q_/_11_ (C-19, cat# sc-392), Gβ (T-20, sc-378) and flotillin-1 (H-104, sc-25506), as well as horseradish peroxidase-conjugated donkey anti-goat IgG (sc-2020), goat anti-rabbit IgG (sc-2004), and goat anti-mouse IgG (sc-2005) antibodies, were purchased from Santa Cruz Biotechnology (Dallas, TX, USA). Mouse monoclonal anti-FLAG antibody (M2, F3165) and mammalian protease inhibitor cocktail was purchased from Sigma (St. Louis, MO, USA). All cell culture reagents were purchased from Life Technologies (Thermo Fisher Scientific, Grand Island, NY, USA). Restriction enzymes and other molecular biology reagents were obtained from Roche Biochemicals (Sigma-Aldrich, St. Louis, MO, USA) or New England Biolabs (Ipswich, MA, USA). OptiPrep was obtained from Axis-Shield PoCAS (Dundee, Scotland, UK). SuperSignal West Pico Chemiluminescent Substrate was purchased from Pierce Biotechnology (Thermo Fisher Scientific, Grand Island, NY, USA). Bio-Rad protein assay dye reagent was purchased from Bio-Rad Laboratories (Hercules, CA, USA). Full-length recombinant PMT and catalytically inactive mutant PMT C1165S were cloned, expressed, purified, quantified, and assayed for biological activity, as previously described [4,21,46].

### 4.2. Mammalian Expression Vectors Encoding Gα Proteins

DNA encoding human Gα_q_, Gα_11_, Gα_q_ Q209L, and Gα_11_ Q209L genes (GB ACC# U43083; UMR cDNA Resource Center) [49] was cloned into the KpnI/XhoI sites of pcDNA3.1^+^ (Invitrogen, Thermo Fisher Scientific, Grand Island, NY, USA). For immunoblot detection, a FLAG-tag flanked at each end by a 6-residue linker sequence (SGGGGS) was introduced between residues 121 and 122 of Gα_q_ and Gα_11_ by using the following primers:
Gα_q_F1: 5′-GCTTGGTACCACCATGACTCTGGAGTCCAT-3′Gα_q_R1: 5′-GAGATATCCGCCGCCGCCAGACACCTTCCCACATCAAC-3′Gα_q_F4: 5′-GAGATATCCCGATACAAAGACGATGACGACAAGGGCGGAGGCGGC-3′Gα_q_F5: 5′-GACGACAAGGGCGGAGGCGGCTCTGCTTTTGAGAATCCATATGTAG-3′Gα_q_R2: 5′-GACTCGAGTTAGACCAGATTGTACTC-3′Gα_11_F1: 5′-GCTTGGTACCACCATGACTCTGGAGTC-3′Gα_11_R1: 5′-GAGATATCCGCCGCCGCCGGACACCTTCTCCACGTCCAC-3′Gα_11_F3: 5′-GACGACAAGGGCGGAGGCGGCTCTACCTTCGAGCATCAGTACGTC-3′Gα_11_R2: 5′-CTAGACTCGAGTCAGACCAGGTTGTAC-3′

(KpnI, EcoRV and XhoI restriction enzyme sites are underlined). All subcloning procedures were confirmed by using restriction enzyme and DNA sequencing analysis. Generation of the Q209E mutants of Gα_q_ and Gα_11_ was completed by PCR cloning using the following primers:
Gα_q_F1: 5′-CGATGTAGGCGGCGAAAGGTC-3′Gα_q_R1: 5′-GACCTTTCGCCGCCTACATCG-3′Gα_q_F2: 5′-TGTCGTAACAACTCCGCCCCA-3′Gα_q_R2: 5′-ACACCTACTCAGACAATGCGATGCA-3′Gα_11_F1: 5′-GTGGGCGGCGAGCGGTCGG-3′Gα_11_R1: 5′-CCGACCGCTCGCCGCCCAC-3′Gα_11_F2: 5′-TGTCGTAACAACTCCGCCCCA-3′Gα_11_R2: 5′-ACACCTACTCAGACAATGCGATGCA-3′

### 4.3. Transient Transfection

HEK-293T cells were maintained in Dulbecco’s modified Eagle’s medium (DMEM) supplemented with l-glutamine and 10% (*v*/*v*) bovine growth serum at 37 °C and 5% CO_2_. Cells grown to 60%–80% confluence (~4 × 10^6^ per 100-mm dish) were transiently transfected for 24 h with mammalian expression vectors: pcDNA3.1 (empty vector), pcDNA3.1/Gα_q_, pcDNA3.1/Gα_11_, pcDNA3.1/Gα_q_ Q209E, pcDNA3.1/Gα_11_ Q209E, pcDNA3.1/Gα_q_ Q209L, or pcDNA3.1/Gα_11_ Q209L, using LipofectAMINE 2000 reagent (Invitrogen), according to the manufacturer’s instructions, or using the CalPhos Mammalian Transfection protocol (Clonetech Laboratories, Takara Bio USA, Mountain View, CA, USA).

### 4.4. Cellular Fractionation

HEK-293T cells, transfected as described above, were treated with or without the indicated amounts of PMT for the indicated times, washed with 1× cold phosphate buffered saline, scraped and transferred into a microcentrifuge tube (Dot Scientific Inc., Burton, MI, USA), then lysed by 10 passages through a 27-gauge needle in 0.5 mL of an ice-cold lysis buffer A (50 mM Tris-HCl, pH 8.0, 2.5 mM MgCl_2_, 1 mM EDTA, 1 mM phenylmethylsulfonyl fluoride, 1 mM benzamidine, 1 mM dithiothreitol, and 10% glycerol) to generate whole cell lysates. Cellular fractionation was performed essentially, as described previously [50]. In brief, nuclei were pelleted by centrifugation at 4 °C and 750× *g* for 5 min, and the post-nuclear supernatant was fractionated into membrane pellets and supernatants by centrifugation at 4 °C and 18,000× *g* for 30 min. The membrane pellets were resuspended in 0.5 mL of lysis buffer A. Membrane proteins were quantified using the Bio-Rad protein assay. Samples containing 10 µg of total membrane proteins were resolved by SDS-PAGE and analyzed by immunoblotting, as described below.

### 4.5. Immunoblotting Analysis

Proteins in samples were separated by 10% SDS-PAGE and transferred onto nitrocellulose membranes, and the membranes were immunoblotted with the indicated primary antibody overnight at 4 °C, washed, then incubated with the appropriate horseradish peroxidase-conjugated secondary antibody. After washing, immunoreactive proteins were visualized by using enhanced chemiluminescence (Pierce Biotechnology, Thermo Fisher Scientific, Grand Island, NY, USA) and captured on autoradiographic film. Image analysis and quantification of protein bands was performed using NIH ImageJ (version 1.48u4, NIH, Bethesda, MD, USA) and Microsoft Excel software (Microsoft Office Professional Plus 2013).

### 4.6. OptiPrep or Sucrose Density Gradient Subcellular Fractionation

DRMs were prepared by density gradient centrifugation with OptiPrep or sucrose, as indicated, using a modification of previously described methods [38,43]. All procedures were carried out on ice. Briefly, four 100-mm culture dishes of subconfluent HEK-293T cells were treated with rPMT at the indicated concentrations for the indicated times. Cells were washed twice with modified Tyrode’s solution (20 mM HEPES, pH 7.4, containing 137 mM NaCl, 2.7 mM KCl, 1.0 mM MgCl_2_, 0.18 mM CaCl_2_, and 5.6 mM glucose). Cells were collected and washed twice with the ice-cold Tyrode’s solution, then centrifuged at 1000× *g* for 10 min. The pellet was solubilized in 1 mL of lysis buffer B (50 mM Tris-HCl, pH 7.4, containing 50 mM NaCl, 0.1% Triton X-100, 5 mM EDTA, 1.0 mM Na_3_VO_4_, 5.0 mM Na_4_P_2_O_7_, and protease inhibitor cocktail, comprised of 0.2 mM aminoethyl-benzene sulfonyl fluoride, 1 µg/mL aprotinin, 10 µM bestatin, 3 µM E-64, 10 µg/mL leupeptin, 2 µM pepstatin, and 50 µg/mL calpain inhibitor I). The cell were lysed via sonication, then incubated at 4 °C for 1 h with frequent agitation. Cell lysates (1 mL) were mixed with 3 mL of OptiPrep or sucrose solution (60% aqueous iodixanol or sucrose, respectively) in a 12-mL centrifuge tube, and the mixture was overlaid first with 4 mL of 35% OptiPrep/sucrose solution diluted in STE buffer (50 mM Tris-HCl, pH 7.6, containing 50 mM NaCl, 5 mM EDTA, and 1.0 mM Na_3_VO_4_,) and then 4 mL of 5% OptiPrep/sucrose solution. Ultracentrifugation was performed at 200,000× *g* and 4 °C for 20 h using a Beckman SW40Ti rotor (Beckman Coulter, Inc., Indianapolis, IN, USA) Fractions of 1.0 mL each were collected from the top of the gradient. Total protein content of each fraction was quantified using Bio-Rad protein assay. The distribution of various proteins in the fractions was assessed by SDS-PAGE and immunoblotting, as described above.

### 4.7. Data Analysis

All data are presented as the mean ± standard deviation (SD) from at least three independent experiments, or as indicated. The *p* values were determined using the unpaired two-tailed Student’s *t*-test. For Figure 4, Figure 5 and Figure 6, two-way ANOVA was also performed using data analysis tools in Microsoft Excel (Microsoft Office Professional Plus 2013) for the curves of control (no toxin treatment) vs. PMT-treated cells over the time course from 0 to 6 h. Statistical significance was set at the 95% confidence level.

## Abbreviations

The following abbreviations are used in this manuscript:

DMEM	Dulbecco’s Modified Eagle’s Medium
DRM	Detergent resistant membrane
EDTA	Ethylenediaminetetraacetic acid
EV	Empty vector
GPCR	G-protein coupled receptor
NT	No transfection and/or treatment
PLCβ1	Phospholipase C β-1
PMT	*Pasteurella multocida* toxin
SD	Standard deviation
SDS-PAGE	Sodium dodecyl sulfate polyacrylamide gel electrophoresis
